# Sunstroke

**Published:** 1898-03-26

**Authors:** 


### SUNSTROKE.

Dr. Sambon puts forward with considerable force the hypothesis that sunstroke or siriasis (as it was called in old times because of its occurrence at those hot seasons when Sirius, the dog star, rises and sets with the sun) is not caused by heat but by infection. It is an ingenious hypothesis, and the facts in favour of it are well marshalled, but it must be understood that it is the wider view which we now take of infections which makes it possible to class such a disease as this among them. In any case, whatever the source of the infection, it seems clear that it only operates in the presence of a high temperature, and the treatment by cold affusion would seem still to be appropriate. The extraordinary prevalence of the disease in certain years and in certain districts, while in other years and other districts quite as hot it is hardly met with, certainly point strongly to some infective or miasmatic origin. Of course, all this refers only to the so-called apoplectic form of the disease, or thermic fever, in which there is hyperpyrexia, profound coma, and congestion of the lungs, and has nothing to do with heat exhaustion, such as affects soldiers marching in close order, or with what is called "stoker's collapse"; these are merely forms of syncope, another affair altogether.

